# Analysis of agency relationships in the design and implementation process of the equity fund in Madagascar

**DOI:** 10.1186/s13104-015-0988-0

**Published:** 2015-02-04

**Authors:** Ayako Honda

**Affiliations:** Health Economics Unit, School of Public Health and Family Medicine, University of Cape Town, Anzio Road, Observatory, 7925 South Africa

**Keywords:** Agency theory, Policy process, User fee, Exemption, Equity fund, Madagascar

## Abstract

**Background:**

There are large gaps in the literature relating to the implementation of user fee policy and fee exemption measures for the poor, particularly on how such schemes are implemented and why many have not produced expected outcomes. In October 2003, Madagascar instituted a user fee exemption policy which established “equity funds” at public health centres, and used medicine sales revenue to subsidise the cost of medicine for the poor. This study examines the policy design and implementation process of the equity fund in Madagascar in an attempt to explore factors influencing the poor equity outcomes of the scheme.

**Methods:**

This study applied an agency-incentive framework to investigate the equity fund policy design and implementation practices. It analysed agency relationships established during implementation; examined incentive structures given to the agency relationships in the policy design; and considered how incentive structures were shaped and how agents responded in practice. The study employed a case-study approach with in-depth analysis of three equity fund cases in Madagascar’s Boeny region.

**Results:**

Policy design problems, triggering implementation problems, caused poor equity performance. These problems were compounded by the re-direction of policy objectives by health administrators and strong involvement of the administrators in the implementation of policy. The source of the policy design and implementation failure was identified as a set of principal-agent problems concerning: monitoring mechanisms; facility-based fund management; and the nature and level of community participation. These factors all contributed to the financial performance of the fund receiving greater attention than its ability to financially protect the poor.

**Conclusion:**

The ability of exemption policies to protect the poor from user fees can be found in the details of the policy design and implementation; and implications of the policy design and implementation in a specific context determine whether a policy can realise its objectives. The equity fund experience in Madagascar, which illustrates the challenges of beneficiary identification, casts doubts on the application of the ‘targeting’ approach in health financing and raises issues to be considered in universal health policy formulation. The agency framework provides a useful lens through which to examine policy process issues.

## Background

User fees for health care services, when combined with appropriate exemption mechanisms, can, in theory, mitigate negative effects on utilisation by the poor as revenue from user fees can be pooled to protect the poor from the requirement to pay for health services and improve health care programmes and facilities targeted at this group [1-3].

However, in practice, many exemption measures for the poor have not succeeded in securing equitable access to health care services [4]. The failure of such measures to protect the poor can be attributed to reasons including: (i) difficulties in accurately identifying the poor; (ii) limited awareness of exemption schemes by the poor; (iii) lack of transparent exemption criteria and procedures which create high transaction costs and uncertainty for those seeking exemptions; (iv) reluctance by providers to grant exemptions, in part due to a lack of incentive for them to do so; and (v) reluctance by the poor to seek exemptions because of social stigma or lack of motivation to use exempted services [5]. In addition, exemption measures can cause significant disruptions to other parts of health systems which are already suffering from a range of resource and capacity constraints [6].

There are large gaps in the literature relating to the implementation of user fee policy and exemption measures for the poor. While a considerable number of studies measure and analyse the impact of user fees and exemption measures on health care service demand and utilisation, few studies examine how exemption schemes are actually implemented and why many of them have not produced the outcomes expected. There is a need, therefore, for studies that examine the implementation process for user fee policy and fee exemption measures for the poor and identify problems that arise in implementation of such policy.

In October 2003, Madagascar instituted a new user fee policy, FANOME (*Fandraisan’Anjara NO Mba Entiko* – Funding for non-stop medicine supplies) that created “equity funds” at public health centres to subsidise free medicine for the poor. The 2005 General Policy of the Government (*la Poliltique Générale de l’Etat: PGE*) stated that 80% of the poor should have access to free medicine through the equity fund scheme. Resources for equity funds are obtained from medicine sales at the community pharmacy attached to public health centres. Management committees, with membership from local communities, operate and manage the funds. Beneficiaries of the fund are identified by local authorities. An assessment of the equity fund policy outcomes indicated that while the exemption system was well targeted, with the mean socio-economic status of equity fund beneficiaries lower than that of non-beneficiaries, both leakage and under-coverage occurred under the scheme and there was failure to protect the majority of those in need from financial burden when accessing health care [7]. This study examines the policy design and the implementation process for the equity fund in Madagascar in an attempt to explore factors influencing the poor equity outcomes of the equity fund.

## Methods

### Study framework

The process of policy development and execution has four elements: agenda setting, formulation, implementation, and evaluation [8]. This process is not necessarily linear [9].

This paper takes the empirical finding that the equity fund in Madagascar failed to protect the poor from the financial costs of illness (a policy evaluation) [7] as its starting point and seeks explanations in terms of how the policy was designed and implemented. The policy design was derived from the formal policy guidelines, and its implementation from how the formal guidelines were executed in practice.

The study uses an agency framework to investigate the policy design and implementation process for the equity fund from the organisational and institutional perspectives. Specifically, the study examines: (1) principal-agent relationships established by the creation of the equity fund; (2) institutional arrangements given to these agency relationships as reflected in the policy design; and (3) how the institutional arrangements were shaped in the course of policy implementation and agents’ responses to the institutional arrangements in the equity fund implementation.

An agency relationship is one in which a person (the agent) acts on behalf of another (the principal). If both parties in the relationship are utility maximisers, the principal and agent can have divergent objectives and, consequently, the agent may not always act in the best interests of the principal. Therefore, the principal must structure incentives so that the agent’s best interests, given those incentives, lead to desirable outcomes for the principal. Incentive structures are shaped by five central elements: resources, information, decision-making, delivery mechanisms and accountability [10]. These elements are used to affect institutional and organisational solutions to principal-agent problems [11]. In this study, these five elements were applied to analyse the institutional arrangements reflected in the policy design and determine how the arrangements were shaped during the operation of the equity funds at health centres in Madagascar.

### Case study

The study employed a case study approach, a preferred strategy when ‘how’ and ‘why’ questions are posed [12], wherein the FANOME equity fund at public health centres was the central unit of analysis. Three equity funds were selected for in-depth analysis based on the following criteria: (1) exemplary and contrasting outcomes in utilisation and income in order to obtain robust theoretical inferences; (2) different health administrative districts in order to examine the principal-agent relationships under different health administrative settings; and (3) different geographical areas to capture variations due to location.

### Study settings

Field data collection was conducted in the Boeny region, in north-western Madagascar, in the period between November 2005 and October 2006. The region includes six health districts and a population of approximately 543,000 people [13].

Case Studies 1 and 2 operate under the same district health administration jurisdiction while Case Study 3 operates under a separate administration. Case Study 1 is located in an urban setting. Although in an urban commune, the location of Case Study 2 demonstrates both urban and rural socio-economic characteristics and is regarded as a suburban setting. The location of Case Study 3 is considered rural.

### Data collection

Data were collected through individual interviews, focus group discussions, structured observation and document review.

A total of 35 individual interviews were undertaken with local health administrators, health service providers, local government authorities and equity fund managers. The interviews were undertaken in Malagasy by a locally-trained interviewer. A list of semi-structured questions developed for the interviews covered a range of issues relating to the equity fund implementation.

A total of 14 focus group discussions were held with community representatives (members of the health committee, CoSan (*Comité de Santé*)), health centre patients and equity fund beneficiaries. Each focus group consisted of seven to eight participants, selected from different geographical areas within each case study district and with consideration of gender and age balance. The focus group discussions were facilitated in Malagasy by a locally-trained facilitator with relevant expertise. A list of discussion topics was prepared to explore how people were informed of, became involved in, and perceived FANOME and the equity fund.

Both the semi-structured interview guide and the focus group discussions topics were prepared in English and translated into Malagasy. To assure translation quality, the translated data collection tools were independently back-translated. Both interviews and focus group discussions were tape-recorded with participant consent.

Structured observations took place in a health centre consultation room and at the community pharmacy, PhaGeCom (*Pharmacie à Gestion Communautaire*), attached to the health centre. The observation monitored how patients, both equity fund beneficiaries and non-beneficiaries, were received by the medical doctor, validated for equity fund status and provided with prescriptions in the consultation room, and the process by which patients were received, validated for equity fund status and provided with medicine at PhaGeCom. In addition, issues relating to equity fund management at PhaGeCom were also examined, including: financial management; management of medicine stocks; and data collection.

For each equity fund case, observation occurred over a one-month period. A midwife was recruited to conduct consultation room observations and a local Non Governmental Organisation (NGO) staff member was employed to conduct observations at PhaGeCom. Both attended the health centre on a daily basis and conducted the observations using a checklist.

The information necessary to document the policy design, context, and implementation was gathered through a review of available literature, reports and policy documentation, primarily sourced from the central government and local health administrators. International donors who were actively involved in the policy formulation process also provided relevant documents and reports.

The study received ethical approval from the ethical committee of Direction Régional de la Santè et du Planning Familial de Boeny, Madagascar (Ministère de la Santé et du Planning Familial) and the London School of Hygiene and Tropical Medicine.

### Data analysis

Audio recordings of interviews and discussions were transcribed verbatim (in Malagasy) into MS-Word documents by locally-trained assistants. To assure transcription quality, transcriptions were independently checked against original audio-recordings. Transcriptions were then translated into French. All of the translated transcriptions were independently checked against the original Malagasy transcriptions to assure the translation quality. French transcriptions and observation reports were later transferred to NVivo 7 for analysis.

This research used both a deductive and an inductive approach to qualitative data analysis. The details of the agency relationships in the equity fund implementation was based on the policy design reported in the “FANOME guide” [14]. The research objectives, the outline of the agency relationships and the five central elements in the institutional arrangements informed the generation of a provisional list of codes prior to coding.

Issues that were not anticipated during the design of the research were explored in depth as they arose during the qualitative data analysis. Thematic analysis was used to identify additional key issues (themes) that emerged from the data and involved three overlapping stages: initial coding; development of a coding scheme; and coding the data. After coding was complete, relationships between the themes were explored.

In order to ensure the reliability of the analysis, a team of local research participants – the individual interviewer, the focus group facilitator and the observer at PhaGeCom – was formed to review the analysis and agree upon the codes and categories used in the analysis.

## Results

In order to understand the equity fund implementation process and explore factors influencing the equity outcomes of the equity funds, findings are presented for: (1) examination of agency relationships established by the creation of the equity funds; (2) analysis of the policy design using the agency framework; and (3) examination of actual practices in the equity fund implementation by looking at the institutional arrangements that were shaped during the policy implementation and agents’ responses to those arrangements during the equity fund implementation.

### Agency relationships in the implementation of equity funds

**Figure 1:**
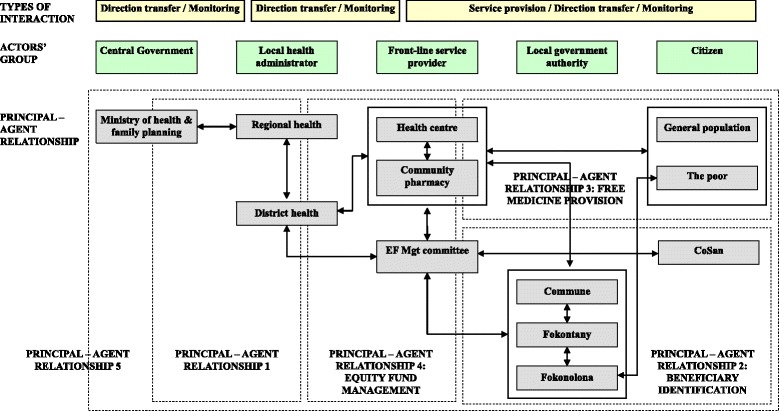
**Outline of agency relationships in the equity fund implementation.**

Instituting equity funds for healthcare in Madagascar established multiple levels of interactions between various actors in the policy implementation process. The actors were classified as: central government; local health administrators; front-line service providers; local government authorities; and citizens. Interactions between the five groups characterise the principal-agent relationship (Figure 1) and include:

- Interactions between central government and local health administrators, and among local health administrators (principal-agent relationship 1);
- Interactions between actors who take part in the identification of eligible beneficiaries (principal-agent relationship 2: beneficiary identification);
- Interactions between actors involved in fee exemption for medicine at community pharmacies (principal-agent relationship 3: free medicine provision);
- Interactions between actors engaged in management of the equity fund (principal-agent relationship 4: equity fund management); and
- Interactions between the government and citizens (principal-agent relationship 5).

The study aims to analyse the equity fund design and implementation process, with the main focus on policy execution. Consequently, analysis of the policy design and the policy implementation process was restricted to principal-agent relationships of those involved in the execution of policy, that is, only those in principal-agent relationships 2, 3, and 4, particularly those at the district level and below rather than extending to all potential principal-agent relationships throughout the system.

Principal-agent relationship 2 concerns those involved in the identification of equity fund beneficiaries. Identification is delegated by the local health administration through the district health office to the local government authorities: the commune, *fokontany* (decentralised village unit) and *fokonolona* (a sub-structure of *fokontany*). Specifically, *fokontany* asks *fokonolona* to identify indigents in their jurisdiction and to submit a list of indigents to *fokontany*. The commune asks *fokontany* to submit a collated list of indigents from each *fokonolona*, with the list validated by the *fokontany* president. Further, the district health office asks the commune to collate the lists of indigents from each *fokontany* in the commune and validate the list as the official list of indigent beneficiaries for the commune. The district health office then assigns the commune the task of distributing the official list back to each *fokontany* and the health centre and to distribute identification documents to equity fund beneficiaries.

Principal-agent relationship 3 involves the delivery of exempted services and/or goods to beneficiaries. Health centre medicine prescribers, either medical doctors or paramedical staff, serve as single agents for beneficiary validation at health centres. Prescribers keep a copy of the indigents list in the consultation room and check whether beneficiary patients who present with identification cards are registered on the list. After validation, prescribers provide beneficiary patients with consultation and examination and prescribe medicine by completing a “*bon de soins,*” that is, a voucher for beneficiaries to receive free medicine. Free medicine provision takes place at PhaGeCom. A dispenser, who distributes medicine at PhaGeCom, is recruited and remunerated by the commune and is supervised by the head of the health centre. The dispenser is required to keep a copy of the indigents list at PhaGeCom and check whether beneficiary patients who present at PhaGeCom are registered on the list. Free medicine is only provided if the name of the person requesting medicine appears on the beneficiary list. The dispenser supplies medicine free of charge to beneficiaries as detailed in the “*bon de soins*”, which has been signed by the prescriber.

Principal-agent relationship 4 involves the management of the equity fund. A management committee, CoGe (Comité de Gestion) is created at each health centre to manage the equity fund. CoGe consists of: (i) a president and treasurer, who are chosen by CoSan; (ii) a representative from the commune, who is appointed by the mayor; and (iii) a representative from the health sector, who is usually the head of the health centre. CoGe are involved in three equity fund management tasks. Firstly, CoGe must open a bank account that is co-signed by the treasurer and the commune CoGe representative. The cheque book is held by the treasurer and the bank record book kept by the commune CoGe representative to enable monitoring of the account. Secondly, the CoGe treasurer must transfer 3/135^a^ of total monthly drug sales revenue from the PhaGeCom account to the equity fund account. The PhaGeCom account is co-signed by the president and the treasurer. Finally, the CoGe treasurer must reimburse the cost of medicine delivered to beneficiary patients from the equity fund account to the PhaGeCom account. CoGe operates under the supervision of the health centre which sends monthly reports on the operation of FANOME and the equity fund to the district health office and the commune.

### Agency problems identified in the policy design

The policy design analysis attempted to identify the formal arrangements in the equity fund scheme by examining the agency relationships, with a particular focus on the principals’ objectives (i.e. the tasks that agents were intended to perform, as indicated in the policy design) and the institutional arrangements that were formed through the establishment of the equity fund (i.e. the arrangements by which the five elements – resources, information, decision-making, delivery mechanisms and accountability – flow between the groups of actors). The analysis also attempted to identify problems in design which hold the potential for problems to arise in the implementation of the equity fund.

#### Beneficiary identification

The principal-agent relationship governing the identification of beneficiaries is characterised by the multiple-agent relationship between the local health administration (principal) and local government authorities (agents), in which the identification and validation process is replicated at different levels of the local administrative hierarchy.

The multiple-agent structure in the policy design makes it difficult for the local health administration (the end principal) to observe the outcome of each of the local government authorities’ (agents) behaviour at the site of beneficiary identification. Consequently, hidden action problems, wherein the agent’s action is not directly observable by the principal and the outcome is affected by the agent’s actions, may arise between local government authorities and the local health administration. The problem for the end principal is how to design institutional arrangements to monitor and influence agents’ behaviour so that the agents perform tasks to fulfil the objectives of the principal.

However, the flow of information in the policy design is not planned in such a way as to allow the local health administration to observe the behaviour of local government authorities at the site of beneficiary identification, making it difficult for the local health administrators to determine whether the beneficiaries identified and validated by the local government authorities meet the necessary requirements to be listed as equity fund beneficiaries. In addition, local government authority agents are expected to undertake identification tasks on top of their everyday workload and without receiving any additional budget, financial or other types of reward. Consequently, the local health administration may have limited ability to influence the behaviour of local administrative agents during beneficiary identification and the agents may be inclined to identify beneficiaries in accordance with their own interests and at the expense of the local health administration.

#### Free medicine provision

The existence of heterogeneous multiple principals – the district health office and the commune – is one of the main features of the principal-agent arrangements for free medicine provision. When principals hold different objectives and interests, they are likely to compete for the agent’s favour [15]. To be more attractive to the agent, the principal needs to structure institutional arrangements to enable the principal to achieve their own goals while also considering the influence of the other principal on the agent. In the case of free medicine provision, the five central elements in the institutional arrangements are mainly designed to flow between the actors in the health sector, although flows of information and accountability exist between the health centre and the commune. Consequently, agents – prescribers at the health centre and dispensers at PhaGeCom – are likely to act in favour of the local health administration principal (the district health office) if conflicts exist in the objectives and interests of the two principals.

The cost recovery aspect of FANOME creates a source of funding for purchasing medicinal stocks and other managerial supplies at both the health centre and PhaGeCom, which implies that health facility staff, including prescribers and dispensers, are not separate from the conflicting interests of providing free medicine to beneficiaries and raising income for facility operations. Failure to separate the interests of health facility staff from the cost recovery aspects of the policy scheme results in potential unwillingness among prescribers and dispensers to grant free medicine to beneficiaries.

Health centres are responsible to both the district health administration and the commune, as outlined in Madagascar’s decentralisation policy. However, the role of the commune in free medicine provision is not clearly specified in the policy design and, therefore, it is possible that the commune will not actively involve themselves in this area of the policy implementation unless there is specific motivation to drive their participation.

#### Equity fund management

The core members of CoGe, that is, the president and treasurer, are elected from the community (CoSan) with approval from the commune. CoGe is assigned managerial tasks by the local health administration and reports to the commune, CoSan and the local health administration on the operation of FANOME and the equity fund. The agency relationship in this area is considered to be a multiple-principal case; CoGe, as an agent manages the equity fund for multiple-principals – the local health administration, the territorial administration (the commune) and CoSan. However, the institutional arrangements between CoGe and each of the principals, and between the principals, in the management of the equity fund lack clarity in formal policy documents, such as the FANOME guide. Specifically, while CoGe is accountable to each of the principals, the majority of the financial resources provided to CoGe do not come from the principals but from patients’ contributions to medicine and family planning purchases at PhaGeCom. The flow of information, both monitoring and guidance provision, is specified between CoGe and the health administration whereas the monitoring mechanisms between CoGe and other principals are loosely defined and remain rather informal.

The main financial resources for operation of the equity fund do not come directly from the principals but from patient contributions to medicine and family planning purchases at PhaGeCom. The CoGe president and treasurer both receive an allowance from this revenue. While the mechanism for the health sector principal to monitor equity fund status is specified within the processes of the local health administration, the commune and CoSan’s roles in monitoring the equity fund are rather loosely defined – The commune representative in CoGe reports to the mayor on CoGe operations. An auditor, who is appointed by CoSan, reviews the financial management of the fund every three months. However, how and the extent to which the commune and CoSan get involved in monitoring activities and how they use the monitoring reports are unclear.

Although some decisions on the use of drug sales revenue are made by CoGe, the fixed rates allocated to medicinal supplies and the equity fund, which account for 80% of drug sales revenue, are predetermined by the Health Ministry. Limited involvement of CoGe in equity fund management was expected to occur due to the loosely defined institutional arrangements between CoGe and each of the principals and the lack of decision-making power for CoGe members over equity fund revenue.

### Actual practices in the equity fund implementation

The agency framework was used to examine how the equity fund policy was actually implemented by comparing the principal’s objectives as described in the policy design and agents’ behaviour in practice, revealing a number of problems relating to agents’ behaviour. The institutional arrangements were considered alongside agents’ behaviour to explore factors influencing the occurrence of problems during beneficiary identification, free medicine provision and equity fund management.

#### Beneficiary identification

As anticipated from the policy design analysis, conflicts of interest between health administrators and village level agents (the actors at *fokontany* and *fokonolona* levels) were found in the process of beneficiary identification. While the district health office and the health centre tried to limit the registration of indigents to a number calculated on the basis of estimated drug sales revenue at PhaGeCom, village level agents were inclined to register more indigents from their own neighbourhoods on the list.

The institutional arrangements given to the principal-agent relationships for beneficiary identification were not shaped in such a way as to direct village level agents to behave in alignment with the end principal’s interests, that is, to balance the number of indigents registered on the list with the amount of money available in the equity fund. Firstly, due to the absence of a mechanism for health administrators to monitor agents’ behaviour at the site of beneficiary identification or *fokontany*’s validation of the list, the district health office and the health centre could only examine the list submitted by *fokontany* as the final output of the village level agents. They could not access the information held by agents or determine how and under what circumstances the village level agents actually identified indigents and validated the list.

Secondly, no particular funding or personal financial rewards were provided for beneficiary identification and validation of the list. Village-level administrators were expected to undertake their equity fund tasks as a part of their everyday work and community participants involved in beneficiary identification in Case Studies 1 and 2, worked as volunteers. Community participants who undertook beneficiary identification expressed strong dissatisfaction with their voluntary status and requested an allowance for their activities. One *fokontany* president explained the situation as:

*…this concerns the motivation of these people who are married and have children and the activities they undertake in observing the population [to identify beneficiaries] take a lot of their time, even days. So I would like these people to be compensated for the period they work as they are poor themselves and yet they must identify the poor. It is as we say in Madagascar “it is just like two blind people holding hands”. These people deserve something.*

(Individual interview with a *fokontany* president)

Although not predicted in the policy design analysis, the equity fund implementation revealed actions taken by the health administration to re-direct the objectives of the equity fund policy. The specific objective of the equity fund announced in PGE in 2005 stated that 80% of the poor should have access to free medicine, however the interpretation of the objective communicated by the Health Ministry to the local health administration focuses more on the amount of funding available in the equity fund being sufficient to cover health care costs associated with 80% of listed beneficiaries than the provision of free medicine to beneficiaries.

*“Provide free medication for 80% of the poor” does not mean that 80% of the poor will become sick and receive consultation at CSB [health centre]. This means that the amount of money available in the equity fund is sufficient to cover 80% of the [listed] indigents in any one year.*

(Directive issued by MINSAN-PF to SSPFD on 16 January 2006, No 09-SANPF/SG/DDDS/SPC)

In this context, concern about limited resources in the equity fund was expressed and the importance of the monitoring financial performance was emphasised.

*Watch carefully so that the fund is not exhausted quickly: set up mechanisms to limit excessive consultations…, organise income generating activities [to supplement the equity fund] with the commune or the region…*

(Directive issued by MINSAN-PF to DRSPF on 10 August 2005, No 310 SAN PF/CAB)

The Health Ministry’s interpretation re-oriented the policy objective by emphasising the financial aspects of the equity fund performance. In accordance with government’s strong promotion of the equity fund’s financial performance, local health administrators, including the district health office and the health centre, were placed under increasing pressure to ensure that sufficient funding was available in the equity fund.

Of major concern to the local health administration was the number of indigents registered on the list compared with the amount of money available in the equity fund. In all of the cases examined, there was a mechanism which allowed the local health administration principal – either the district health office or the health centre, or both – to influence the number of indigents registered on the list before the commune provided final approval of the list. This is a deviation from the beneficiary identification mechanism reflected in the policy design, wherein identification of indigents and validation of lists were to be undertaken within the local territorial administration. In Case Studies 1 and 2, after *fokontany* submitted the list and before the commune gave final approval, the list went through the district health office and the health centre where they discussed the number of beneficiaries and whether or not the equity fund could support all of the listed indigents considering the estimated drug sales revenue at PhaGeCom. In Case Study 3, before the commune validated the list, the health centre checked the list and tried to limit the number of beneficiaries. In all of the case studies, the health administrators, the end principals, appeared to have de facto decision-making power over the number of beneficiaries registered on the list.

Village level agents were not happy about the removal of names from the list and considered it a source of conflict.

*…there were 18 names on my list, and when it came here [to health centre], they said that they would only take four. And so she [the doctor] said there is a limit…I reduced the list to four, because I know the very poor people…and then the people [the doctor] returned after that, “three”, she said, and I reduced it to three…There are three now…This is now creating conflict with the fokonolona.*

(Focus group discussion with *fokontany* presidents)

In all case studies, village level agents were accountable to local health and territorial administrators for beneficiary identification and validation of the list. The commune, in turn, was accountable to the district health office, the health centre and *fokontany* for the provision of the officially validated list. There was no formal structure to inform agents at the *fokonolona* level of the final list approved by the commune.

In Case Studies 1 and 2, beneficiaries on the final list were informed of their status during the identification process and/or during the distribution of identification cards. Those who were removed from the final list were not informed of their removal and learned that they were not on the list when they accessed the health centre. In Case Study 3, identification cards seemed to be mistakenly distributed to elderly people and not to beneficiaries.

The scheme was not publicly announced, consequently, most of the beneficiaries were unaware of the benefits they were entitled to receive and no clear information on the scheme was provided to non-beneficiaries. *Fokontany* presidents hesitated to publicise the scheme. One *fokontany* president said that everyone was equally poor and if the existence of the fund was announced, many people would come to the *fokontany* office with inquiries about how to ensure inclusion on the indigents list. In fact, in spite of the lack of publication of the scheme, *fokontany* received a number of queries from those who were not listed seeking to ascertain why they were excluded.

*There is no communication [with the citizens about the equity fund] because everyone is vulnerable. And once you make a statement, the fokontany will be overwhelmed…But we cannot make an official announcement for the poor, because everyone is poor.*

(Individual interview with a *fokontany* president)

#### Free medicine provision

Prescriber agents appeared to set limits on claims for free medicine by trying to confirm whether listed indigents who presented at the consultation room were actually poor or by strictly forbidding beneficiaries from receiving free medicine more than twice. Consequently, a number of beneficiary patients did not receive free medicine when they accessed the health centre. While health centre staff believed this process necessary as they considered that some beneficiaries did not appear to be poor, beneficiaries who did not receive free medicine at the health centre felt disappointed in the scheme and often complained to the village level agents.

*…“You lie!” they say to you…“One made us buy drugs there, you say that we do not have to pay, but they made us buy drugs. And the small amount of money we have to buy meat goes on drugs.”*

(Focus group discussion with CoSan)

As revealed in the policy design analysis, the policy did not separate the interests of health facility staff from the cost recovery aspects of the scheme. Health centres had no own-budget and drug sales revenue provided funding to purchase medicinal stocks and managerial tools for the health centre and PhaGeCom. Obtaining a secure source of funding for operations was crucial for health centres to effectively manage medicine stocks, consumable items, and managerial tools. Failure to cover these costs meant restricting the functions of the facility. To health centre staff, granting free medicine meant a monetary loss of revenue for the centre and PhaGeCom. Therefore, it was not surprising that prescribers became reluctant to provide free medicine, particularly when facilities already faced financial constraints.

In addition, the mechanism for monitoring free medicine provision was set solely within the health administration, whose main concern was that the reserves in the equity fund be sufficient to cover expenses associated with 80% of listed beneficiaries. Consequently, strong emphasis was placed on monitoring the financial performance of the equity fund, which may have led prescribers to become more vigilant about the balance between free medicine provision and the reserves available in the equity fund.

Along with the re-orientation of the policy objectives, the Health Ministry sent a series of directives instructing health centres to disallow equity fund beneficiaries from receiving free medicine more than twice and not to provide free medicine costing over Ar 1,000. (If the prescription cost more than Ar 1,000, equity fund beneficiaries were required to purchase the medicine themselves). Although health centre staff thought that the need to discourage frequent visits by equity fund beneficiaries was inevitable due to the limited resources in the equity fund, they found the directives difficult to follow. If a beneficiary patient had a severe illness or injury, Ar 1,000 was often insufficient to cover the cost of the medicine required for treatment.

*Because they (the Health Ministry) determined that the expenditure on a (beneficiary) patient must be only Ar 1,000 while a patient has, say quite a simple wound…can you give him some Amox[icillin]? A packet of Amox costs Ar1,150. And so the patient cannot have the packet. And if he [his condition] also gets worse, he has fever, for example, then we cannot give him paracetamol anymore. This is a simply strange situation due to the latest rule.*

(Individual interview with CSB staff)

In Case Study 3, prescribers often performed no validation during consultations with beneficiary patients. It seemed that the prescriber recognised beneficiaries personally. While most beneficiaries were unaware of the benefits they could receive at the health centre, there were some beneficiaries who were aware of their equity fund status and who made frequent visits to the health centre. In order to continue to provide free medicine (more than twice), the head of the health centre gave these people a prescription under the name of a beneficiary who did not often visit the health centre. The health centre appeared to be concerned about frequent visits by beneficiaries. They thought that once beneficiary patients became accustomed to receiving free medicine, it was difficult to convince the patients to start paying again. This departure from correct procedure was possible due to the absence of a formal mechanism allowing the district health office to monitor the behaviour of prescribers at the site of validation. Consequently, prescribers held de-facto decision-making power over whether patients should receive free medicine.

Limited involvement of the commune representatives occurred in all of the case studies which, as indicated in the analysis of the policy design, is attributed to the fact that the institutional arrangements between the commune and the health sector are unclear – while accountability mechanisms exist between the local health administrators and the commune on the performance of the equity fund, resources only flow within the health sector, prescribers held decision making power and monitoring mechanisms existed between health centres and the local health administration.

#### Equity fund management

The management of the equity fund was affected by a participation constraint wherein limited involvement of CoGe members was observed. In addition, correct financial management procedures were not undertaken and strong involvement of the health centre in fund management was observed.

Participation constraints were found among CoGe members, particularly in Case Studies 1 and 3. In Case Study 1, the CoGe president only participated in the management of medicine stocks at PhaGeCom and the treasurer and the commune CoGe representative seldom came to the health centre. In Case Study 3, the newly appointed CoGe president had not decided yet whether he would accept the position, while the CoGe treasurer only worked at the health centre twice a month. The commune CoGe representative rarely participated in equity fund-related activities. In Case Study 2, although CoGe participated in the management of both medicine stocks and sales revenue at PhaGeCom, the head of the health centre kept all the managerial tools with her and CoGe was unable to operate without her.

In addition to the unclear contractual relationships between CoGe and each of the principals, the institutional arrangements, particularly those relating to resources, information and decision-making, can further explain unwillingness by CoGe to participate in equity fund managerial tasks. In Case Studies 1 and 2, the CoGe president and treasurer received an allowance while other CoGe members, commune representatives and health centre staff, were expected to perform their tasks as a part of their everyday work. While the allowance appeared insufficient to motivate presidents and treasurers to participate in the equity fund management, the commune CoGe representative may have felt it unfair that they had to undertake the same activities as the president and treasurer without receiving an allowance. In Case Study 3, where no allowance was provided to any of the CoGe members, the president and treasurer clearly expressed discontent with their volunteer status and complained about the lack of financial incentives to participate.

*…there is no salary eh! Even if there are people who want to do the work, if there is no salary then why should I waste my time there? And I thought this: What if nobody wants to do it? And so I will do it but it is only voluntarily, so I will not spend too much time there but do it once every two weeks. That is why it is done every two weeks…*

(Individual interview with a CoGe member)

Accompanying the inadequate and inequitable financial incentives, CoGe was not given any decision-making power over the use of revenue from drug sales – the source of the equity fund. The fixed share allocated for the purchase of medicinal supplies and the equity fund contribution was predetermined by the Health Ministry and accounted for 80% of the drug sales receipts. Therefore, even in the policy design, the decision-making power given to CoGe was limited. Furthermore, in the equity fund operation, the heads of the district health office and the health centre appeared to retain the de-facto decision-making power over the allocation of funds, including over the remaining 20% of the drug sales revenue over which, according to the policy design, CoGe was supposed to have discretion. Consequently, the CoGe’s tasks in the financial management of the equity fund were restricted to administrative chores, such as signing forms or preparing supporting documents for monthly reports.

*Indeed, the expenditure – such as for the purchase of medicine, transportation expenses, purchase of management tools – are recorded by both the head of the CSB [health centre] in a notebook that she keeps with her, as well as by the treasurer. The head of CSB authorises all the FANOME-related expenses. The treasurer only takes care of the disbursements and filing of supporting documents.*

(Observation at PhaGeCom)

These deviations from correct management procedures offered the opportunity for strong involvement by the health centre in the management of the equity fund with a variety of divergences from the guidelines. For instance, in Case Study 2, as the newly elected CoGe treasurer did not often visit the health centre, the health centre head and the pharmacy dispenser were temporarily undertaking the treasurer’s duties. The pharmacy dispenser kept the medicine sales revenue in the safe at PhaGeCom and, on the 15th of every month, the health centre head deposited the revenue into the bank account of the family planning program (not the PhaGeCom account). This practice was justified as allowing sales revenue to be safely kept in the bank and enabling the health centre head to withdraw money from the bank account whenever it was necessary to purchase medicinal supplies without having to rely on the co-signatories (the CoGe treasurer and the commune CoGe representative), who were frequently absent.

Although the equity fund scheme was designed to separate the authority and funding from health facility staff by entrusting the fund management to CoGe, in practice, the health centre was undertaking a large proportion of the equity fund managerial tasks.

In addition, the monitoring mechanism determined by the health administration emphasised the financial performance of the equity fund – the district health office regularly checked monthly monitoring reports which specified income, expenditure and the balance in the equity fund. Failure to record sufficient funding indicated poor performance in the operation of the equity fund. The health centre might have responded to such a performance monitoring mechanism by actively participating in fund management and making sure that appropriate financial information was recorded in the monthly reports submitted to the district health office. The reports did not include mechanisms to allow the district health office to monitor the behaviour of the health facility staff and CoGe members in their equity fund management activities.

## Discussion

The analysis of the policy design identified a number of shortfalls that had the potential to cause problems in the implementation of the equity fund. In each case study, most of the problems anticipated to occur from the examination of the policy design actually occurred in the implementation of the policy. These problems concerned monitoring mechanisms, facility-based fund management and the level and nature of community participation.

Additionally, issues emerged in relation to actions taken by the health administrators which re-orientated the policy objective after the commencement of the equity fund implementation, changing the emphasis of the equity fund operations to favour financial performance. In line with the re-orientation of the policy objective, the flow of the five central elements of the institutional arrangements was altered in such a way as to allow intervention by the health administration principals to direct the policy implementation to favour their own interests.

Accordingly, the implementation is not serving the goal of the equity fund policy, that is, to improve access to health care for the poor through protection from the financial burden of paying user fees. The main concern of the health administration principal is that the amount of funding available in the equity fund be sufficient to cover the health care costs associated with 80% of listed beneficiaries, rather than providing the country’s indigents with access to appropriate health care (or the utilisation of the scheme by beneficiaries).

Little evidence exists on the success of exemption mechanisms to improve access to health care for the poor and reduce financial burden on the poor [4]. Targeting, the identification of the poorest or those most in need of exemption, has proven in practice to be a major challenge [16-20]. Furthermore, the failure of many exemption schemes has been partly attributed to provider reluctance to grant exemptions when conflicted between granting exemptions and raising income, as exempted patients cause a loss of revenue for facilities already under financial stress. Consequently, “third-party payer” systems, including health equity funds, have been created in several countries in Asia and Africa as an alternative exemption approach that incorporates a mechanism to ensure sufficient funding is available at health facilities and to compensate facilities for the cost of granting exemptions so that the health provider can achieve both equity and efficiency [17,21]. Some reviews of this type of mechanism suggest that adequate health facility or health administration funding from external sources; an independent agent determining eligibility of beneficiaries based on clear, agreed criteria; and regular updates of the beneficiary list are all key to the success of the mechanism but pre-existing health services must also be operating with sufficient financial and human resources [17,21,22]. Nonetheless, there are large gaps in the knowledge relating to the process of implementing fee exemption measures for the poor at the ground-level, particularly about how such schemes are actually implemented and why many schemes have not produced the outcomes intended. This paper addresses those knowledge shortfalls.

The study highlights a number of issues which require attention from those at the policy-making level. Predominantly, the results suggest that, in order to enable a policy to realise its objectives in a particular context, careful attention should be paid to addressing the details of the policy design and implementation that can influence policy outcomes.

The study results also provide useful information from ground-level that should be considered by policy makers. Firstly, the equity fund experience in Madagascar illustrates the potential risk of simply applying a successful public policy ‘model’ in a different context. The health equity fund is a pro-poor health care financing model that has been successful in other settings in terms of effectively improving access to health care for the poor through a reduction in financial burden [17,21]. The results of this study suggest that the application of health care financing policy which has been successful in other settings must be accompanied by a well-planned policy design and deliberate implementation strategies with careful consideration given to contextual factors if the policy is to achieve its goals and objectives. When applying a ‘successful’ case in other settings, the policy development process should carefully look at the actors who will be involved in the policy implementation, the capacity of those actors to carry out the necessary tasks and the relationships between the actors in addition to giving due consideration to the health system and other contexts in which the policy is being introduced. This process should identify potential constraints to policy implementation and enable a clear implementation strategy to be developed to address the potential problems. In the course of implementing the policy, careful, on-going monitoring of the implementation on the ground and adjustment or amendment of the policy design should be undertaken to address unexpected issues. This requires shared policy objectives between health administrators at both the central and local levels and strong commitment by all involved to meeting the policy objectives.

Secondly, there has been on-going debate on whether ‘universalism’ or ‘targeting’ should be the principle underpinning the provision of social policy. Whereas ‘universalism’ considers the entire population to be the beneficiary of social benefits, ‘targeting’ involves approaches such as means testing or proxy indicators to determine the eligibility of individuals for social benefits [18]. Empirical evidence reveals, however, that for health care financing, targeting, that is the identification of the poorest or those most in need of exemption from user fees, has proven, in practice, to be a major challenge [23], and that the approach entails high administrative costs and significant administrative sophistication and capacity [18].

The equity fund experience in Madagascar, which employed a community approach to target benefits, casts doubts on the application of ‘targeting’, demonstrating the complex nature of beneficiary identification, which resulted in a divergence of interests among those involved in identifying poor households. The village-level agents were inclined to register too many indigents from their own neighbourhoods on the beneficiaries list, while the district health office and the health centre tried to limit the number of indigents on the basis of estimated drug sales revenue at PhaGeCom. More fundamentally, the socio-economic context of Madagascar, where the poverty rate is nearly 70%, poses questions as to whether it is appropriate to employ a ‘targeting’ approach when most of the population suffers from poverty.

Lastly, the study results suggest dominance of the health sector (end principal) in the management of the equity fund and difficulty in obtaining genuine participation from the community. In addition to the inadequate financial incentives offered to the community CoGe representatives, no decision-making power was given over drug sales revenue, the source of the equity fund. Instead, the heads of the district health office and the health centre retained decision-making power over the allocation of funds. Consequently, CoGe’s tasks in the financial management of the equity fund were limited to administrative chores. The study results imply that in order to achieve effective community participation, careful consideration needs to be paid to the roles of community participants and other actors involved in the policy execution and the degree to which decision-making authority, resources and collective tasks are shared.

Using the agency framework to examine the policy design helped identify problems that could be anticipated to arise in the policy implementation. In fact, many of the agents’ behaviour-related problems that actually occurred in the equity fund implementation were identified as principal-agent problems and were predicted to arise due to limitations in the policy design.

In addition to identifying problems in the policy design, the agency framework was useful in the analysis of policy implementation practices to identify problems relating to the behaviour of actors involved in the policy implementation. Major problems in agents’ behaviour were explained by how the five central elements in the institutional arrangements, i.e. resources, information, decision-making, delivery mechanisms and accountability, were structured in the principal-agent relationships.

However, the agency framework used in this study is not without limit. As this paper aims to analyse the design and implementation process of the equity fund, the main focus of the study is policy execution. Therefore, the scope of this study is restricted to the principal-agent relationships of those who undertake tasks related to the equity fund implementation, particularly those at the district level and below. Consequently, the possibility exists that important factors outside the scope of this study may have influenced the policy outcomes but these factors were not included in the analysis. Resolution of this problem requires expansion in the scope of the agency framework to encompass other relationships in the policy formulation and implementation, for example, the relationship between the central government and local health administrators and between the government and citizens.

Few studies have applied agency theory to analysing health systems in low and middle income countries [24-27], although a number of studies have applied principal-agency theory to analysing health care management in higher income countries [28-31]. More studies utilising agency analysis are required to further understand the relevance and contribution of the agency perspective to health systems research.

Lastly, the data collection undertaken in this study took place several years ago and the author understands the reader’s concerns about the ‘freshness’ of the information and the discussion provided in this paper. However the author strongly believes that the study results: (1) contain invaluable messages from ‘on-the-ground’ experience of implementing a pro-poor health care financing policy in Madagascar, one of the least developed countries in Africa; (2) contribute to filling gaps in the literature relating to how user fee policy and exemption measures for the poor are actually implemented and why many of them have not produced the outcomes expected; and (3) present an innovative use of an agency framework to investigate the design and implementation of the equity fund policy in Madagascar.

## Conclusion

This study used agency theory to confirm that shortfalls in the policy design, the re-orientation of the policy objective and the re-shaping of institutional arrangements in the implementation of the equity fund in Madagascar all contributed to poor equity performance of the equity fund. The study results provide warning of potential risks in simply employing a successful public policy ‘model’ in a different context and suggest that, in order to determine whether a policy can realise its objectives, it is necessary to ascertain the implications of the policy design and implementation in a particular context. The equity fund experience in Madagascar, which illustrates the challenges of beneficiary identification, feeds into international debate on ‘universalism’ and ‘targeting’ in health financing and raises issues to be considered in the formulation of universal health policy. Lastly, effective community participation requires careful consideration of the roles, resources and decision-making authorities shared by the community participants and other actors.

## Endnote

^a^At PhaGeCom, medicines are sold to patients at a margin of 35% of the price fixed at the national level [14].
